# The influence of adrenoceptor blocker treatment on fracture healing in osteoporotic and non-osteoporotic bone

**DOI:** 10.3389/fphys.2025.1726583

**Published:** 2026-02-16

**Authors:** Sandra Dieterich, Christoph Kölbl, Miriam Eva Angelica Tschaffon-Müller, Oliver Küppers, Dorothea Gebauer, Melanie Rebecca Kuhn, Katarina Teresa Acker, Anita Ignatius, Melanie Haffner-Luntzer

**Affiliations:** 1 Institute of Orthopaedic Research and Biomechanics, University Medical Center Ulm, Ulm, Germany; 2 Department of Orthopaedic Trauma, University Medical Center Ulm, Ulm, Germany

**Keywords:** adrenergic signaling, adrenoceptor blocker treatment, butoxamine, fracture healing, inflammation, phentolamine, postmenopausal osteoporosis, propranolol

## Abstract

Fracture healing is a highly dynamic process that involves inflammation, cell recruitment, angiogenesis, and subsequent bone formation and remodeling. Increasing evidence suggests the pivotal role of adrenergic signaling in musculoskeletal repair and bone-related diseases such as osteoporosis. Furthermore, impaired fracture healing in osteoporotic female mice might be attributed to an overshooting immune response with increasing numbers of neutrophils found in the early fracture hematoma. Earlier studies showed that an unspecific blockade of the β-adrenoceptor with propranolol reduces the number of neutrophils in the fracture hematoma in male mice, which might also help alleviate the overshooting immune response in female osteoporotic mice. In this study, we hypothesized that adrenoceptor blocker treatment in the early inflammatory phase of fracture healing rescues the excessive immune response in osteoporotic female mice and thereby improves fracture healing. However, our results indicate that an early blockade of adrenergic receptors does not improve fracture healing in osteoporotic and non-osteoporotic mice. In contrast to earlier studies with male mice, beta blockade (propranolol and butoxamine) in female non-osteoporotic mice increased the number of neutrophils in the early fracture hematoma, indicating an elevated immune response and a sex-dependent effect of adrenoceptor blocker treatment.

## 1 Introduction

Bone fracture healing is a complex process and has three overlapping phases—inflammation, repair, and remodeling phases (Claes et al., 2012). The fracture incidence itself leads to tissue trauma with blood vessel damage, formation of a hematoma, and subsequent immune system activation. The early inflammatory phase is dominated by neutrophils that are recruited to the initial fracture hematoma to remove the cell debris by phagocytosis and attract other immune cells such as macrophages, monocytes, and lymphocytes (Kovtun et al., 2016). Additionally, mesenchymal and endothelial precursor cells are recruited, which then proliferate and differentiate to enable proper fracture healing. During the repair phase, first, a cartilaginous callus is formed, which is then replaced by a more stable bony callus (Claes et al., 2012; Einhorn and Gerstenfeld, 2015). In the subsequent remodeling phase, the original bone contour and structure are restored (Claes et al., 2012). Bone possesses a high natural ability for regeneration, with the possibility of scarless healing. However, several comorbidities such as age, drugs, or diseases can lead to impaired fracture healing (Kostenuik and Mirza, 2017). Osteoporosis is associated with increased fracture risk and delayed fracture healing (Nikolaou et al., 2009). Osteoporosis is characterized by low bone mineral density caused by altered bone microstructure (Porter and Varacallo, 2025; Varacallo and Fox, 2014). The most common form is postmenopausal osteoporosis due to the decrease of estrogen in women after menopause, which leads to higher bone resorption compared to bone formation (Avioli, 1976). In addition to an endogenous decline in the healing capacity, fracture fixation is also more challenging in osteoporotic bone; therefore, new treatment strategies are urgently needed (Burge et al., 2007). Recently, it has become evident that the innervation of the bone with sensory and sympathetic nerve fibers plays a crucial role during fracture healing (Shi et al., 2021; Niedermair et al., 2020). In addition, there is growing evidence that local catecholamine signaling, particularly adrenergic signaling, may play a role in the pathomechanism of osteoporosis (Ma et al., 2013). Catecholamines (adrenaline, noradrenaline, and dopamine) are mainly secreted in the adrenal glands upon stimulation of the sympathetic nervous system and can bind to different subtypes of adrenoceptors (often also called adrenoreceptors), namely, α_1_, α_2_, β_1_, β_2_, and β_3_ (Khalil et al., 2025). They are crucial for various physiological processes, such as stress response and cardiovascular function (Khalil et al., 2025). In addition, catecholamines are also synthesized locally in myeloid immune cells as a response to chronic stress and might cause impaired fracture healing (Kuhn et al., 2022; Tschaffon-Muller et al., 2023). Catecholamines are known to mediate negative stress effects on bone healing by activating α/β_2_ adrenoceptors on chondrocytes, thereby compromising their trans-differentiation toward osteoblasts and resulting in impaired bone fracture healing (Kuhn et al., 2022; Tschaffon-Muller et al., 2023). Other studies reported that α_1_ signaling is beneficial for osteoblast proliferation and differentiation, while β_2_ signaling showed the opposite effect (Huang et al., 2009; Takeda et al., 2002; Hedderich et al., 2020). This is also indicated in osteoblast-specific *Adrb2*-KO mice and general *Adrb2*-KO mice showing enhanced bone mass (Elefteriou et al., 2005; Pierroz et al., 2012). In 2021, Yokomoto-Umakoshi and colleagues demonstrated that catecholamine-producing tumors increase the risk of osteoporosis and osteoporotic fractures (Yokomoto-Umakoshi et al., 2021), while patients treated with the unspecific β-blocker propranolol benefited from its osteoprotective effects (Bonnet et al., 2008). An overshooting immune response by increased neutrophil numbers in female osteoporotic mice was reported by Haffner-Luntzer et al. (2017), while male non-osteoporotic mice treated with propranolol immediately before fracture displayed a significantly lower number of neutrophils in the early fracture hematoma (Haffner-Luntzer et al., 2019). This indicates that elevated levels of neutrophils in the early inflammatory phase of fracture healing might contribute to impaired fracture healing in osteoporotic mice (Haffner-Luntzer et al., 2017). Early short-term blockade of adrenoceptor signaling could, therefore, have a positive effect on fracture healing, especially in osteoporotic bone.

We performed RNA-sequencing of fracture callus tissue obtained from osteoporotic and non-osteoporotic mice, in which adrenergic signaling was detected as a potential pathomechanism of delayed osteoporotic fracture healing. Furthermore, we specifically analyzed the effects of different adrenoceptor blockers, namely, propranolol (unspecific β-blocker), butoxamine (specific β_2_-blocker), and phentolamine (unspecific α-blocker), which were injected during the early inflammatory phase of fracture healing, on fracture healing in osteoporotic and non-osteoporotic bone.

## 2 Materials and methods

### 2.1 Study approval

All animal experiments were performed in accordance with good scientific practice, in compliance with international regulations for the care and use of laboratory animals (ARRIVE), and with the approval of the local ethical committee (license 1612, Regierungspräsidium Tübingen, Germany).

### 2.2 Animals, surgical procedures, and euthanasia

**FIGURE 1:**
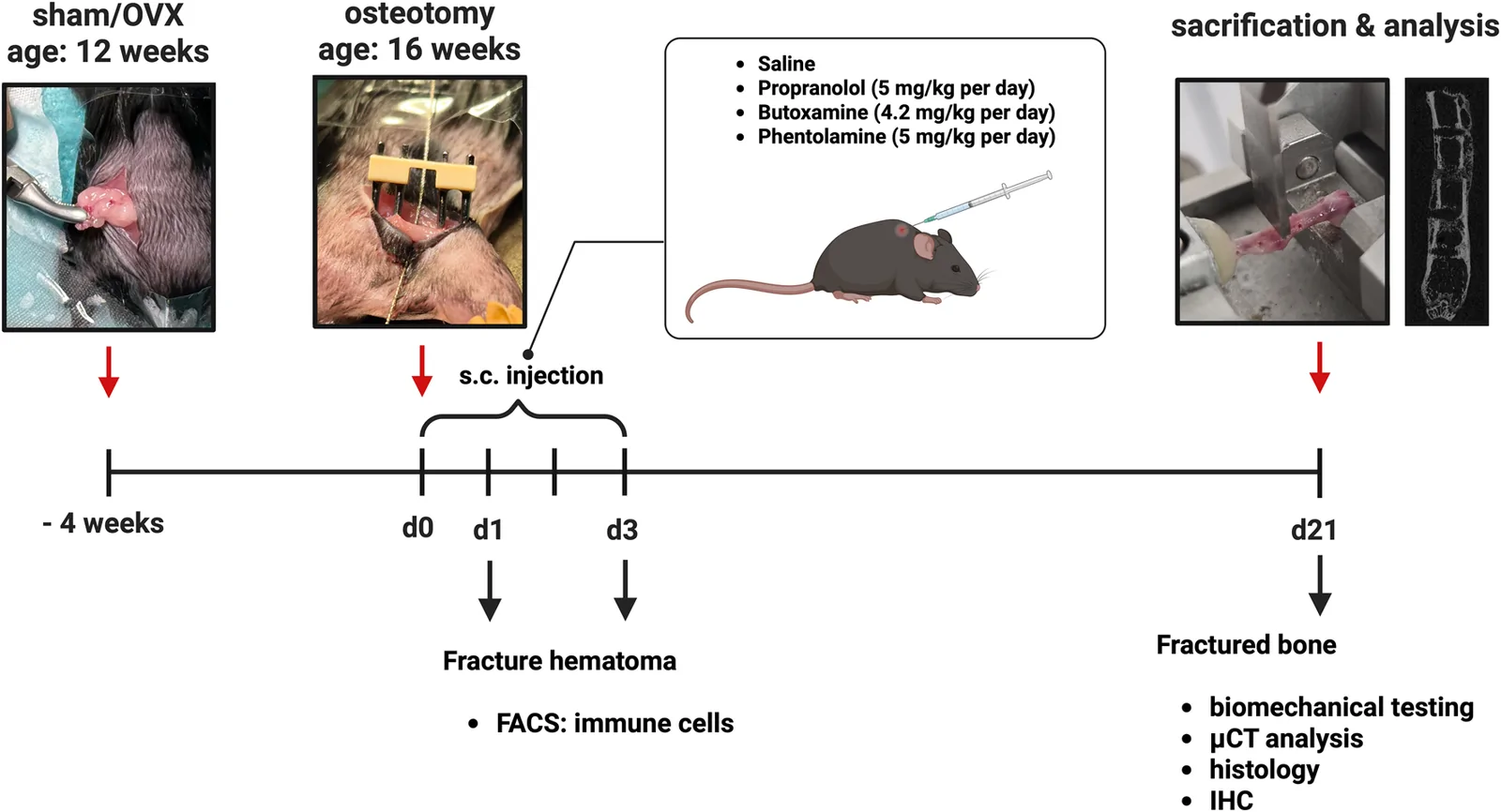
Timeline of the adrenoceptor blocker study (created in BioRender. Ohmayer, W (2026) https://BioRender.com/zwoluxc).

The study workflow is depicted in Figure 1. Female C57BL/6J mice aged 6–8 weeks were purchased from Charles River Laboratories (Sulzfeld, Germany) and housed in groups of up to four per cage under a 12-h light/dark cycle with *ad libitum* access to food and water. The animals were randomly distributed into different treatment groups. At the age of 12 weeks, a bilateral ovariectomy (OVX) or sham surgery (sham) was performed. Four weeks after sham/OVX surgery, the mice received standardized unilateral femur osteotomy stabilized with a semi-rigid external fixator (RISystem, Davos, Switzerland). Osteotomy was generated with a 0.44-mm Gigli wire saw (RISystem) at the femur diaphysis. For pain treatment, the animals were provided tramal hydrochloride (0.1 mg/mL) in the drinking water at 1 day pre-surgery and for 3 days post-surgery. On the day of surgery, anesthesia was performed with 5–6% isoflurane and maintained with 2% isoflurane with an oxygen flow rate of 0.7 mL/min. Directly before surgery, the mice received a subcutaneous injection of 500 µL sterile saline, clindamycin (45 mg/kg BW), and tramal hydrochloride (25 mg/kg BW). At different timepoints post-osteotomy (1, 3, and 21 days), the mice were euthanized in the morning (8 a.m.–10 a.m.) with isoflurane overdose and subsequent blood withdrawal from the heart. Biomechanical testing, µCT analysis, and histological analysis were performed after 21 days. Samples collected after 1 or 3 days post-surgery were used for flow cytometry. Additionally, the blood plasma (stored at -80 °C) was used for multiplex cytokine enzyme-linked immunosorbent assay (ELISA) and 3-CAT ELISA.

### 2.3 Adrenoceptor blocker administration

On the day of fracture surgery and 3 days post-surgery, the mice received either a subcutaneous injection of the vehicle solution (NaCl) or an adrenoceptor blocker (propranolol 5 mg/kg, butoxamine 4.2 mg/kg, or phentolamine 5 mg/kg) once daily (Figure 1).

### 2.4 Multiplex ELISA

Cytokine levels in the blood plasma were determined using a ProcartaPlexTM Mouse Immune Monitoring Panel (48-Plex) from Invitrogen (EPX480-20834-901). Concentrations of BAFF, BTC, CXCL5, eotaxin (CCL11), G-CSF (CSF3), GM-CSF, Gro-α (CXCL1), IFN-α, IFN-γ, IL-1α, IL-1β, IL-10, IL-12p70, IL-13, IL-15, IL-17A (CTLA 8), IL-18, IL-19, IL-2, IL-22, IL-23, IL-25 (IL 17 E), IL-27, IL-28, IL-2R, IL-3, IL-31, IL-33, IL-33R (ST2), IL-4, IL-5, IL-6, IL-7, IL-7R-α, IL-9, IP-10 (CXCL10), leptin, LIF, M-CSF, MCP-1 (CCL2), MCP-3 (CCL7), Mip-1α (CCL3), Mip-1β (CCL4), Mip-2α (CXCL2), RANKL, RANTES (CCL5), TNF-α, and VEGF-A were measured.

### 2.5 3-CAT ELISA

The Fast Track 3-CAT ELISA from LDN Nordhorn, Germany (BA E−6600), was used to measure the catecholamine levels in the hematoma, bone marrow, and blood. Adrenaline, noradrenaline, and dopamine levels were calculated and analyzed with the Arigo biolaboratories ELISA calculator.

### 2.6 Biomechanical testing

To assess the fracture healing outcome, the flexural rigidity of the fracture callus compared to that of the unfractured femur was tested using a non-destructive three-point-bending test 21 days after fracture surgery. In a material testing machine (Zwick Roell, Ulm, Germany), the fractured bone without a fixator was loaded with up to a maximum value of 2 N, and the load and deflection were recorded. The flexural rigidity was calculated with the slope of the load–deflection curve, as described previously (Haffner-Luntzer et al., 2016). In total, five to eight mice were analyzed per group.

### 2.7 µCT analysis

After biomechanical testing, the femora were fixed in 4% paraformaldehyde for 48 h until further histological processing. Within this time, the femora were scanned using the SkyScan 1172 (Bruker, Kontich, Belgium) (50 kV, 200 mA, 8 µm). The bones were analyzed with the CTanalyzer and CTvolume software applications (Bruker). Two phantoms with defined hydroxyapatite content (250 and 750 mgHA/cm3) were used to calculate the bone mineral density. The callus was defined as the region between the two inner pinholes. Tissue mineralization was defined as above a threshold of 642 mg HA/cm3. In addition, the contralateral unfractured femur was analyzed. The threshold for trabecular bone was set above 394 mg HA/cm3, while the cortical parameters were analyzed with a threshold of 642 mg HA/cm3. Five to eight mice were analyzed per group.

### 2.8 Histomorphometry

After biomechanical testing and CT scanning, the bones were subjected to decalcified histology. Sections were stained with Safranin O and tartrate-resistant acid phosphatase (TRAP) to evaluate the amount of bone, cartilage, and fibrous tissue, as well as bone forming (osteoblast) and bone resorbing (osteoclast) parameters. The bone sections 21 days after fracture surgery were analyzed with light microscopy based on an image-analysis software (Leica DMI6000B, Software MMAF 1.4.0 MetaMorph, Leica, Wetzlar, Germany). The region of interest chosen was between the inner pins, including the periosteal callus and the fracture gap. The whole callus, bone, and cartilage were framed, while the fibrous tissue was calculated by subtracting the bone and cartilage from the total callus. For illustration, the results are depicted as the percentage of the whole callus. With the help of the OsteoMeasure system (OsteoMetrics, Decatur, GA, USA), the evaluation of osteoblasts and osteoclast parameters within a rectangular area (650 × 450 μm) in the middle of the periosteal fracture callus was done. Five to eight mice were analyzed per group.

### 2.9 Flow cytometry

On 1 and 3 days post-fracture, immune cell populations were quantified in the hematoma, bone marrow of the left unfractured femur, and spleen using flow cytometry, as described previously (Haffner-Luntzer et al., 2017). The samples were collected and processed for flow cytometry analysis. After erythrolysis, the samples were centrifuged (5804 R, Eppendorf) for 5 min at 1800 rpm at room temperature in 10 mL phosphate-buffered saline (PBS; Thermo Fisher Scientific). Hundred microliters of each sample was added to a round-bottomed 96-well plate (650180, Greiner Bio-One) and incubated for 30 min on ice in the dark with fluorescently labeled antibodies (rat anti-mouse F4/80 FITC 1:50, 11-4801-82, eBioscience; CD3e PE-Cyanine7 1:100, 25-0031-82, eBioscience; rat anti-mouse CD8a APC 1:800, 17-0081-81, eBioscience; rat anti-mouse CD4 APC-eFluor 780 1:200, 47-0041-82, eBioscience; rat anti-mouse CD11b Alexa Fluor 700 1:400, 56-0112-80, eBioscience; rat anti-mouse CD19 PE 1:400, 12-0193-81, Affymetrix, eBioscience; rat anti-mouse Ly6G V450 1:400, 560603, BD Biosciences). As isotype controls the anitbodies rat IgG2A, rat IgG2a κ FITC (1:50, 11-4321-82, eBioscience), Armenian hamster IgG PE-Cyanine7 (1:100, 25-4888-82, eBioscience), rat IgG2a κ APC (1:800, 17-4321-81, eBioscience), rat IgG κ APC-eFluor 780 (1:200, 47-4031-82, eBioscience), rat IgG2a κ Alexa Fluor 700 (1:400, 56-4031-80, eBioscience), rat IgG2a κ PE (1:400, 12-4321-81, Affymetrix, eBioscience), and rat IgG2a κ V450 (1:400, 560377, BD Bioscience) were used. After incubation, the samples were washed in FACS buffer, transferred into FACS vials together with 100 µL 7AAD (1:100 in FACS buffer), and then measured. CD11b^+^ myeloid immune cells, Ly6G^+^ neutrophils, F4/80^+^ macrophages, CD3e^+^ T lymphocytes, CD3e^+^CD8a^+^ cytotoxic T lymphocytes, and CD3e^+^CD4^+^ T helper lymphocytes were quantified with the KI-based unbiased clustering software application Cytolution (Fischer et al., 2025a) using the gating strategy, as described previously (Fischer et al., 2025b).

### 2.10 RNA-sequencing

Callus tissue from OVX and sham mice at day 14 after fracture surgery was dissected, minced, and fresh-frozen. RNA was isolated, and RNA-seq analysis was performed by Novogene GmbH, as described previously (Haffner-Luntzer et al., 2023). Differentially regulated gene (DEG) identification and GO and KEGG analyses were performed as described previously (Haffner-Luntzer et al., 2023). RNA-seq data were uploaded to Zenodo (doi: 10.5271/zenodo.17812737).

### 2.11 Immunohistochemistry (IHC) of ADRA1 and ADRB2

Longitudinal paraffin sections of 4 µm thickness were prepared for IHC staining of the adrenoceptor alpha 1 (ADRA1) and adrenoceptor beta 2 (ADRB2). For the detection of ADRA1, the primary antibody rabbit α alpha1AR (1:100; antikörper-online.de; ABIN2854646; polyclonal IgG) was incubated overnight at 4 °C. For the detection of ADRB2, the primary antibody rabbit α beta2AR (1:100; BIOSS, bs-0047R, polyclonal IgG) was incubated overnight at 4 °C. For both kinds of single staining, the secondary antibody goat anti-rabbit IgG-biotin [1:200 (ADRA1); 1:1000 (ADRB2); B2770, Life Technologies] was applied at room temperature (RT) for 1 h. Horseradish peroxidase (HRP)-conjugated streptavidin (PK-6100, VECTASTAIN Elite ABC-HRP Kit, Peroxidase, Vector Laboratories) was used for signal detection following the manufacturer’s instructions. NovaRED (SK-4800, Vector NovaRED Substrate Kit, Peroxidase (HRP), Vector Laboratories) served as the chromogen, and sections were counterstained with hematoxylin (1:2000; 2C-306, Waldeck). Species-specific, non-targeting immunoglobulins were used as isotype controls.

### 2.12 Statistical analysis

**FIGURE 2:**
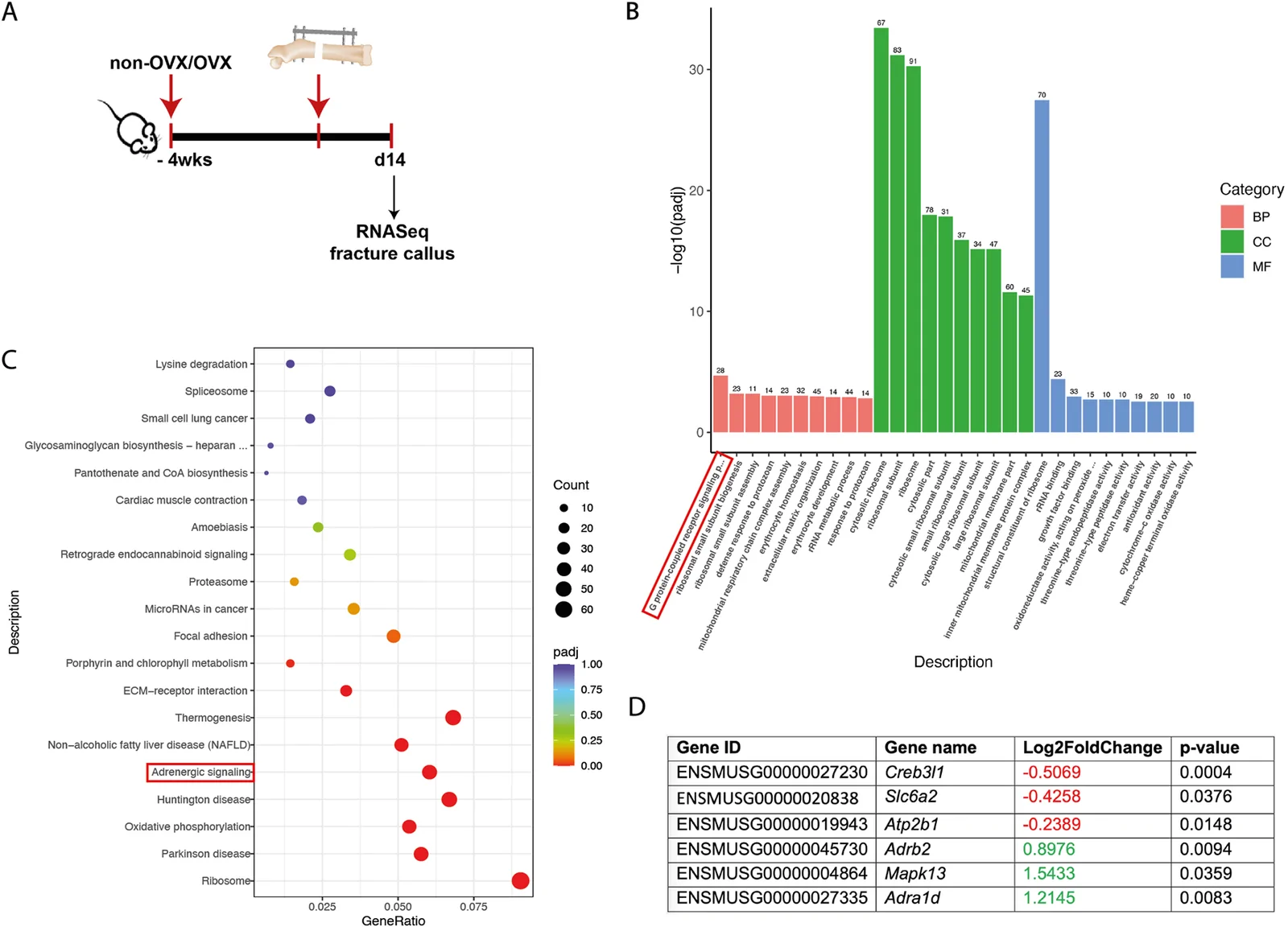
RNA-sequencing analysis of fracture callus tissue from non-OVX versus OVX mice at day 14 after fracture. **(A)** Experimental design. **(B)** GO term analysis of significantly regulated genes in non-OVX vs. OVX mice displayed in the subcategories “biological process” (BP), “cellular component” (CC), and “molecular function” (MF). Number of regulated genes and -log10 (padj) are shown. **(C)** KEGG pathway analysis from significantly regulated genes in non-OVX vs. OVX mice. The abscissa is the ratio of the number of differential genes linked with the KEGG pathway to the total number of differential genes. The ordinate is the KEGG pathway. The size of a point represents the number of genes annotated to a specific KEGG pathway. The color from red to purple represents significant enrichment level. **(D)** Significantly up- or downregulated genes related to adrenergic signaling in the fracture callus of non-OVX vs. OVX mice (*N* = 3-4).

Statistical analysis was performed with Graph Pad Prism 10.0 software (GraphPad software, La Jolla, CA, USA). Data gained from the 3-CAT ELISA (Table 1) were tested with the unpaired t-test and with ordinary two-way ANOVA with uncorrected Fisher’s LSD *post hoc* test (Figure 2). Flow cytometry data, P1NP-ELISA, and fracture healing data, including biomechanical testing, µCT, and histology, were tested with ordinary two-way ANOVA with uncorrected Fisher’s LSD *post hoc* test. IHC staining data (Supplementary Figure 1) were tested with unpaired t-test. Data in figures and tables are depicted as bars with mean ± standard deviation. The level of significance was set at p < 0.05. The group size was 3–8.

**TABLE 1:** Catecholamine measurements of pre-fracture, day 1, and day 21 post-fracture. Adrenaline, noradrenaline, and dopamine in ng/mL.

Catecholamines	Type Time-point	Blood Pre-fracture	Blood Day 1	Blood Day 21	Unfractured femur Pre-fracture	Unfractured femur Day 1	Unfractured femur Day 21	Fractured femur Day 1	Fractured femur Day 21
Adrenaline	Sham	**7.61 ± 2.07**	3.39 ± 2.77	7.60 ± 2.08	0.10 ± 0.03	0.09 ± 0.03	**0.03 ± 0.02**	0.03 ± 0.02	0.03 ± 0.02
	OVX	**5.33 ± 1.17** ^**T**^	6.33 ± 2.95	6.54 ± 1.32	0.08 ± 0.02	0.08 ± 0.03	0.07 ± 0.01*	0.04 ± 0.02	0.05 ± 0.02
Noradrenaline	Sham	**6.11 ± 2.61**	**1.62 ± 0.64**	6.10 ± 3.99	0.72 ± 0.29	**0.90 ± 0.23**	0.56 ± 0.24	0.32 ± 0.17	0.10 ± 0.02
	OVX	3.42 ± 0.61*	**4.08 ± 2.27** ^**T**^	4.46 ± 1.31	1.00 ± 0.49	**1.26 ± 0.32** ^**T**^	0.58 ± 0.32	0.49 ± 0.29	0.12 ± 0.06
Dopamine	Sham	**0.52 ± 0.20**	0.33 ± 0.25	0.69 ± 0.33	0.14 ± 0.05	0.12 ± 0.02	0.10 ± 0.03	0.06 ± 0.06	0.03 ± 0.03
	OVX	0.27 ± 0.05*	0.36 ± 0.22	0.45 ± 0.14	0.17 ± 0.14	0.15 ± 0.08	0.09 ± 0.04	0.07 ± 0.05	0.05 ± 0.04

Statistical significance was determined by unpaired t-test (comparison of sham vs. OVX per timepoint). T = 0.05–0.10, *P < 0.05, **P < 0.01, ***P < 0.001, and ****P < 0.0001. (N = 3-6).

Bold values highlight observed differences between sham and OVX mice.

## 3 Results

**FIGURE 3:**
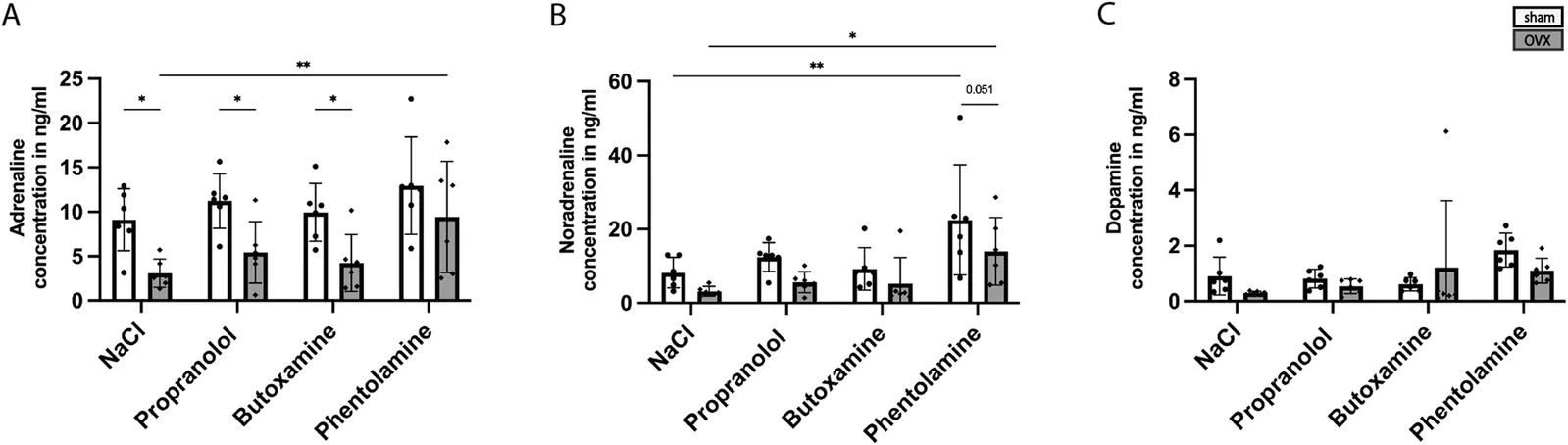
Catecholamine measurement in the blood plasma at 3 days post fracture. Adrenaline concentration in ng/mL; **(A)** noradrenaline concentration in ng/mL; **(B)** and dopamine concentration in ng/mL **(C)** in NaCl-, propranolol-, butoxamine-, and phentolamine-treated sham and OVX mice. Statistical significance was determined by two-way ANOVA with Fisher’s LSD test. *P < 0.05, **P < 0.01, ***P < 0.001, and ****P < 0.0001 (*N* = 6).

RNA-sequencing of fracture callus tissue from sham and OVX mice at 14 days post-fracture surgery revealed a significant regulation of genes within the GO term G-protein-coupled receptor signaling (Figures 2A,B). KEGG pathway analysis showed differentially regulated genes within adrenergic signaling, including the adrenoreceptor β_2_ (*Adrb*2) and adrenoreceptor α_1D_ (*Adra1d)* (Figures 2C,D). The expressions of both genes were significantly upregulated in the fracture callus of OVX mice at 14 days post-fracture (*Adrb2* = 0.8976-log2fold; *Adra1d* = 1.2145-log2fold). However, on protein level, immunohistochemistry staining of the Adrb2 and Adra1 in the fracture callus at 14 and 21 days post-fracture revealed no difference in adrenoceptor expression, which might be because of time-delayed protein expression (Supplementary Figure 1) (Acker, 2025). To further elucidate the potential role of adrenergic signaling, catecholamine levels were measured in the blood samples, fracture calli, and bone marrow of the intact contralateral femora of the control and OVX mice at different timepoints (Table 1). Pre-fracture OVX mice showed decreased levels of adrenaline and significantly decreased levels of noradrenaline and dopamine in the plasma. At 1 day post-fracture, the levels of noradrenaline in the blood plasma and unfractured femur were increased in OVX mice. At 21 days, post-fracture adrenaline levels were significantly higher in OVX mice. In the fractured femur, no differences could be detected at 1 and 21 days post-surgery (Table 1). Additionally, blood samples were collected 3 days post-surgery to assess the catecholamine levels upon blocker treatment (NaCl, propranolol, butoxamine, and phentolamine). Figure 3A demonstrates that adrenaline levels were significantly decreased in NaCl-, propranolol-, and butoxamine-treated OVX mice compared to the respective sham group. Phentolamine treatment in OVX mice led to a significant increase in adrenaline levels compared to NaCl-treated sham mice. Noradrenaline levels were also increased in phentolamine-treated sham and OVX mice compared to NaCl-treated sham and OVX mice (Figure 3B). Dopamine concentrations did not differ with blocker treatment (Figure 3C).

**FIGURE 4:**
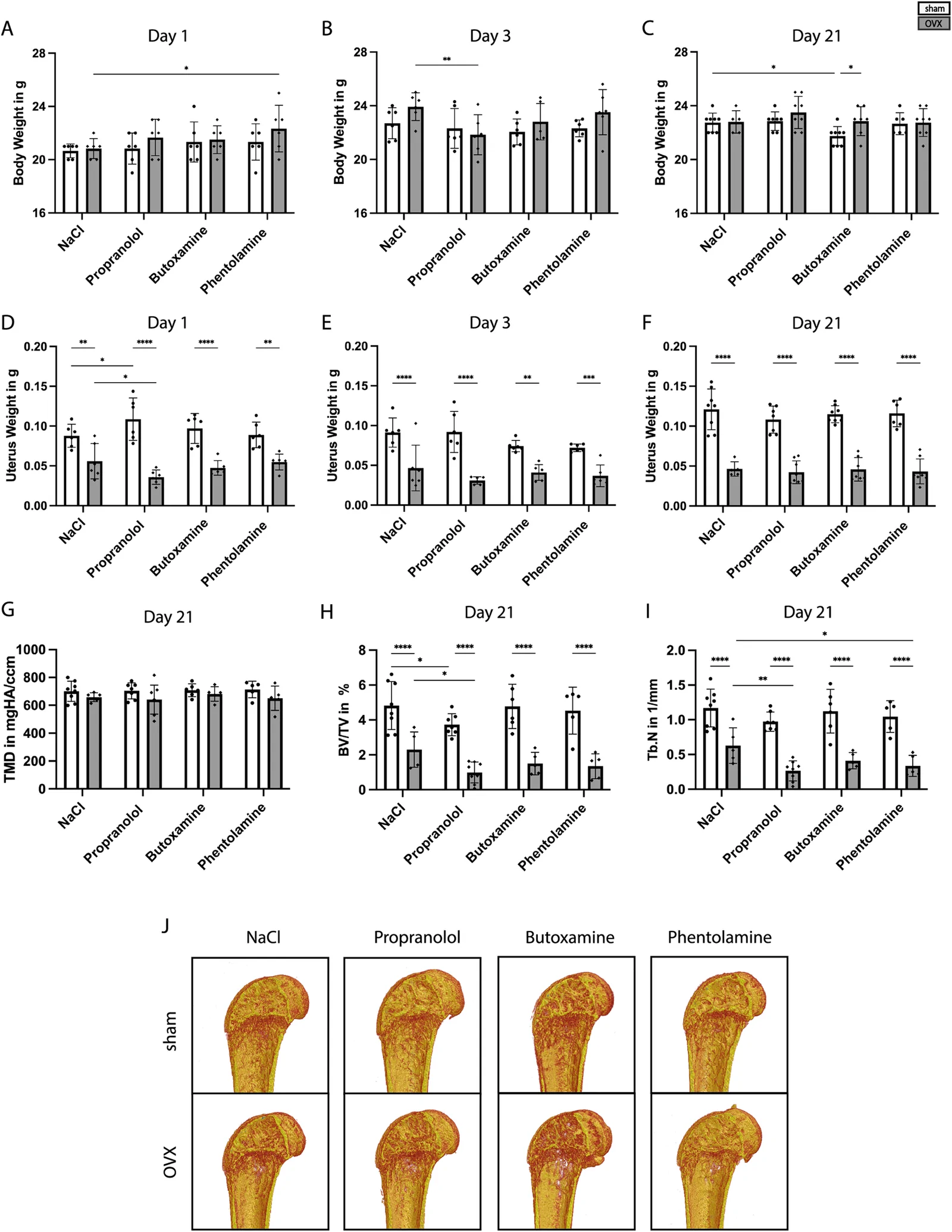
Analysis of mouse bodyweight, uterus weight, bone volume per tissue volume (BV/TV in %), and trabecular number of the unfractured left femur. **(A–C)** Bodyweight of mice in g on day 1 **(A)**, day 3 **(B)**, and day 21 **(C)**. **(D–F)** Uterus weight of mice in g on day 1 **(D)**, day 3 **(E)**, and day 21 **(F)**. **(G)** Tissue mineral density (TMD) of the trabecular bone. **(H)** Trabecular BV/TV in % of the unfractured left femur (day 21 mice). **(I)** Trabecular number (Tb.N) in 1/mm of the unfractured left femur (day 21 mice). **(J)** 3D reconstruction of the trabecular bone volume in the left unfractured femur (day 21 mice). Statistical significance was determined by two-way ANOVA with Fisher’s LSD test. *P < 0.05, **P < 0.01, ***P < 0.001, and ****P < 0.0001 (*N* = 5-8).

C57BL/6J female mice were treated with the adrenoceptor blockers propranolol (unspecific β-blocker), butoxamine (specific β_2_-blocker), and phentolamine (unspecific α-blocker) in the first 3 days after fracture surgery to gain new insights into the role of adrenergic signaling in the early phase of fracture healing in both osteoporotic and non-osteoporotic bone. The mice were randomly distributed to different treatment groups, with bodyweight measurements showing no major between-group differences (Figures 4A–C). To confirm successful ovariectomy, the uterus was collected and weighted. A significant reduction in uterus weight for all OVX mice could be seen (Figures 4D–F). Osteoporosis was successfully induced, indicated by no differences in the tissue mineral density (TMD) but reduced bone volume/tissue volume (BV/TV) and trabecular number (Tb.N) (Figures 4G–I) in the unfractured femur. Figure 4J presents the 3D reconstruction images of the unfractured femur at 21 days post-surgery, further demonstrating trabecular bone loss in OVX mice compared to sham mice.

**FIGURE 5:**
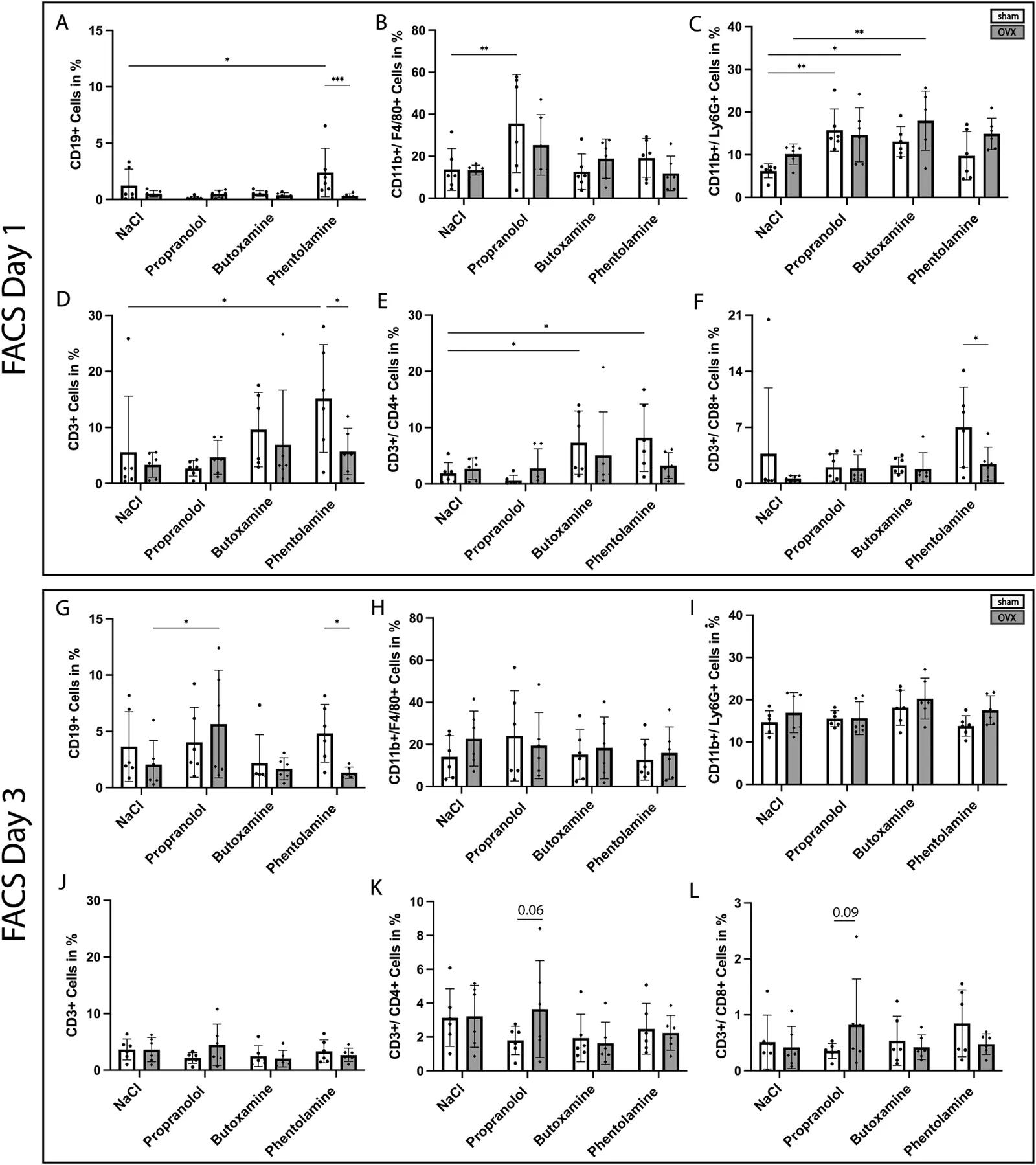
FACS-analysis of the hematoma at 1 day and 3 days after fracture surgery. **(A–F)** Percentage of CD19^+^ **(A)**, CD11b^+^/F4/80^+^ **(B)**, CD11b^+^/Ly6G^+^ **(C)**, CD3^+^ **(D)**, CD3^+^/CD4^+^ **(E)**, and CD3^+^/CD8^+^ **(F)** cells in the fracture hematoma at 1 day post-surgery. **(G–L)** Proportion of CD19^+^ **(G)**, CD11b/F4/80^+^ **(H)**, CD11b^+^/Ly6G^+^ **(I)**, CD3^+^ **(J)**, CD3^+^/CD4^+^ **(K)**, and CD3^+^/CD8^+^ **(L)** cells in the fracture hematoma at 3 days post-surgery. Analysis was performed with the KI-based software Cytolution. Statistical significance was determined by two-way ANOVA with Fisher’s LSD test. *P < 0.05, **P < 0.01, ***P < 0.001, and ****P < 0.0001 (*N* = 6).

Flow cytometry was performed to evaluate the effects of the different adrenoceptor blockers on immune cell recruitment during the early inflammatory phase of fracture healing. Immune cells were analyzed as the proportion in % of all living cells. At 1 day post-fracture, phentolamine treatment led to differences in immune cell recruitment. The comparison of sham and OVX phentolamine-treated mice revealed a significantly reduced proportion of CD19^+^, CD3^+^, and CD3^+^/CD8^+^ cells in phentolamine-treated OVX mice (Figure 5, FACS day 1, A, D, F). Phentolamine treatment in sham mice led to a significantly higher proportion of CD19^+^, CD3^+^, and CD3^+^/CD4^+^ cells compared to sham control (NaCl) mice (Figure 5, FACS day 1, A, D, E). Additionally, propranolol- and butoxamine-treated sham mice displayed a significant increase in CD11b^+^/Ly6G^+^ cells compared to sham control mice, which could also be detected for butoxamine-treated OVX mice compared to OVX control mice (Figure 5, FACS day 1, C). Distinct effects could only be seen upon blocker treatment at 3 days post-surgery (Figure 5, FACS day 3). Phentolamine-treated sham mice showed an increase in CD19^+^ cells compared to phentolamine-treated OVX mice, while CD19^+^ cells were increased in propranolol-treated OVX mice compared to OVX control mice (Figure 5, FACS day 3, G). The parent populations of F4/80^+^ and Ly6G^+^ cells on day 1 and day 3 after fracture are depicted in Supplementary Figures S2A,B.

**FIGURE 6:**
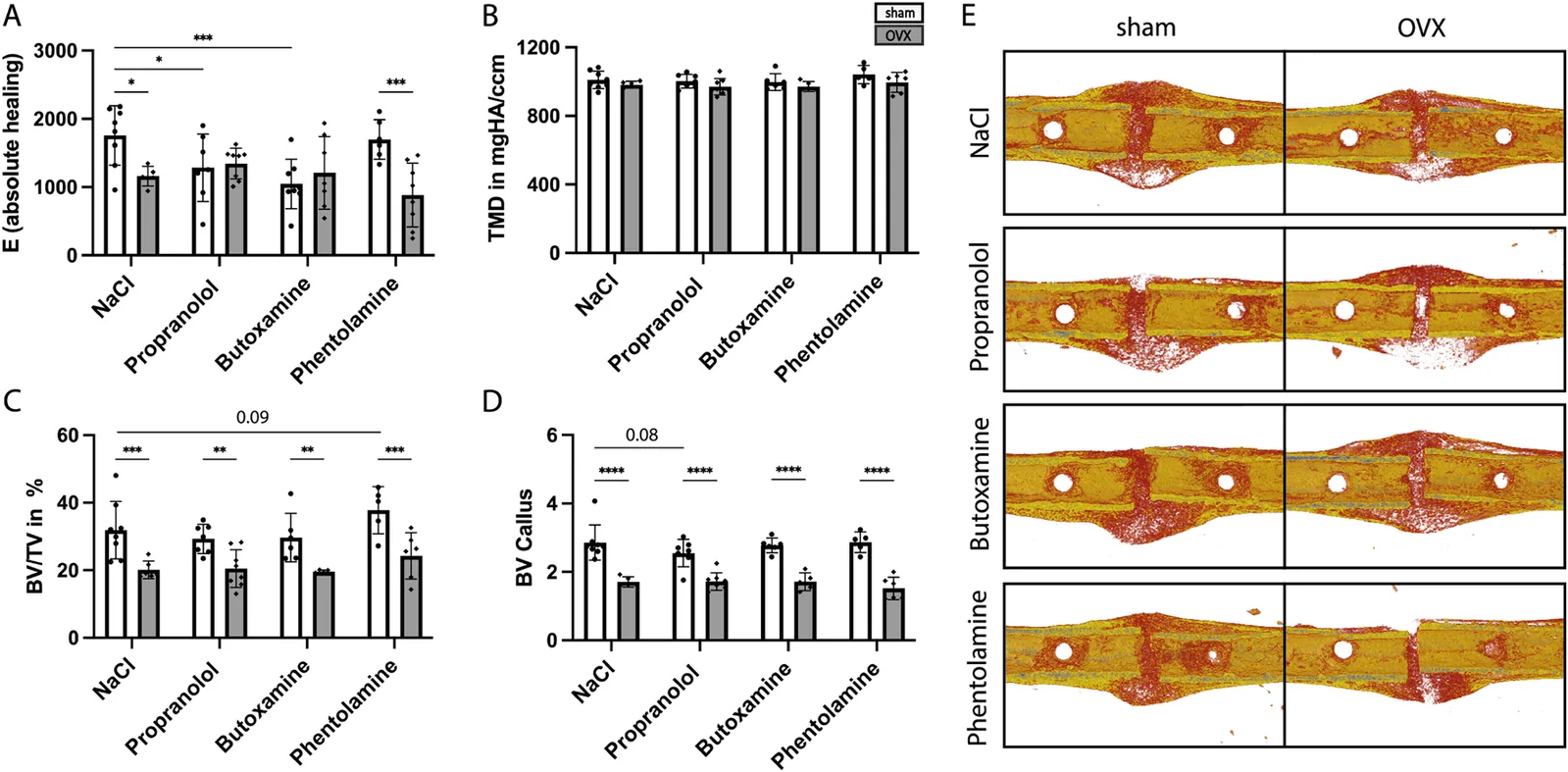
Biomechanical testing and µCT analysis at day 21 after fracture surgery. **(A)** Absolute healing (E in 1/mm). **(B)** Tissue mineral density (TMD) in mgHA/ccm of the fracture callus. **(C)** Bone volume per tissue volume (BV/TV in %) of the newly formed callus. **(D)** Bone volume (BV) of the fracture callus. **(E)** 3D-reconstruction images of the fracture callus from one representative mouse of each treatment group. Statistical significance was determined by two-way ANOVA with Fisher’s LSD test. *P < 0.05, **P < 0.01, ***P < 0.001, and ****P < 0.0001 (*N* = 5–8).

**FIGURE 7:**
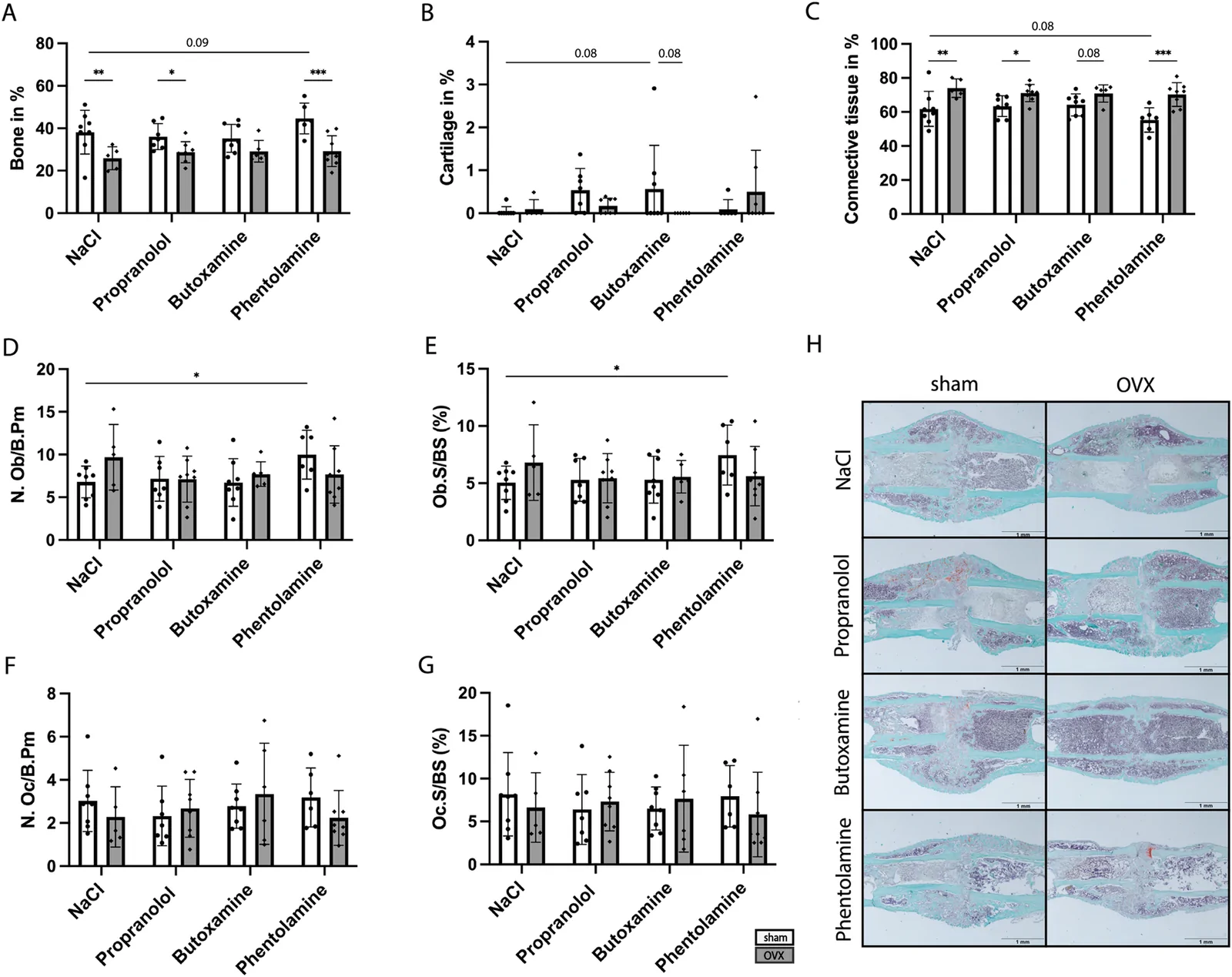
Histomorphometry at day 21 after fracture surgery. **(A–C)** Percentage of bone **(A)**, cartilage **(B)**, and connective tissue **(C)** in the newly formed callus. **(D,E)** Analysis of the number of osteoblasts per bone perimeter **(D)** and osteoblast surface per bone surface **(E)**. **(F,G)** Analysis of the number of osteoclasts per bone perimeter **(F)** and osteoclast surface per bone surface **(G)**. **(H)** Safranin-O/fast green staining of the fracture callus from a representative mouse of each treatment group. Dark blue = bone marrow; light blue = bone; red: cartilage. Statistical significance was determined by two-way ANOVA with Fisher’s LSD test. *P < 0.05, **P < 0.01, ***P < 0.001, and ****P < 0001 (*N* = 5–8).

Twenty-one days post-surgery, the mice were sacrificed to assess the overall fracture healing outcome upon blocker treatment. The analysis of the 3-point-bending test revealed significantly reduced absolute bending stiffness in NaCl- and phentolamine-treated OVX mice compared to sham mice, while no difference was detected for propranolol- and butoxamine-treated sham and OVX mice (Figure 6A). Furthermore, absolute bending stiffness was significantly reduced in propranolol- and butoxamine-treated sham mice compared to sham control mice (Figure 6A). µCT analysis showed no difference in the TMD independent of every treatment (Figure 6B) but a significantly decreased BV/TV (Figure 6C) and a significant reduction in the bone volume (BV) (Figure 6D) in OVX mice compared to the respective sham group, indicating delayed healing in osteoporotic mice in all treatment groups. 3D reconstruction images of the representative mice of each treatment group demonstrate that blocker treatment could not improve callus formation (Figure 6E). The histological evaluation results confirmed these data (Figure 7). Bone content was significantly reduced, cartilage unaffected, and connective tissue was increased in the OVX control and propranolol- and phentolamine-treated mice compared to the respective sham group (Figures 7A–C). Osteoblast and osteoclast analyses showed no differences between the sham and OVX mice (Figures 7D–G), but osteoblast activity is significantly increased in phentolamine-treated sham mice compared to sham control mice (Figures 7D,E). No differences in P1NP, a marker for osteoblast function, could be observed between the groups (Supplementary Figure 3). Figure 7H shows the representative images of the evaluated callus tissue of every treatment group stained with Safranin-O/fast green. Bone tissue is shown in light blue, cartilage tissue is shown in red, and bone marrow is shown in dark blue.

## 4 Discussion

Adrenergic signaling, initiated by binding of catecholamines to adrenergic receptors, has been increasingly associated with disturbances in bone homeostasis and fracture healing. Bone cells such as osteoblasts, osteoclasts, osteocytes, and chondrocytes express adrenergic receptors, namely, α_1_, α_2_, β_1_, β_2_, and β_3_, mediating different effects (Huang et al., 2009; Aitken et al., 2009; Opolka et al., 2012). It has been shown that α_1_ signaling is beneficial for osteoblast proliferation and differentiation, while β_2_ signaling showed the opposite effect (Huang et al., 2009; Takeda et al., 2002; Hedderich et al., 2020). This is evident in osteoblast-specific *Adrb2*-KO mice and general *Adrb2*-KO mice displaying enhanced bone mass (Elefteriou et al., 2005; Pierroz et al., 2012). Additionally, immune cells such as neutrophils express adrenergic receptors and are influenced by catecholamines (Trabold et al., 2007). Kuhn et al. reported that myeloid cells are capable of producing catecholamines by themselves, thereby influencing bone turnover and regeneration in mice (Kuhn et al., 2022). Proper immune-cell recruitment is essential for successful fracture healing in the early inflammatory phase (Claes et al., 2012). Previous studies from our group have shown that the number of neutrophils was increased in the early fracture hematoma of female osteoporotic mice (Haffner-Luntzer et al., 2017) and in male mice suffering from chronic stress (Haffner-Luntzer et al., 2019), while blockade of adrenergic signaling with the unspecific beta blocker propranolol was able to rescue the latter effect, further highlighting the potential role of adrenergic signaling in fracture healing (Haffner-Luntzer et al., 2019). We analyzed the potential pathomechanisms of delayed fracture healing, assessed systemic catecholamine levels, and treated female mice with different adrenoceptor blockers in the early inflammatory phase of fracture healing to analyze their effect on fracture healing in osteoporotic and non-osteoporotic bone. With this administration of different adrenoceptor blockers in the early inflammatory phase, we wanted to ensure that we only address the immune cells directly after fracture. The influence of adrenergic signaling on immune cell recruitment and the subsequent impact on the overall fracture healing outcome was thus analyzed.

To assess potential pathomechanisms of delayed fracture healing, we performed RNA-sequencing from fracture callus tissue from non-OVX and OVX mice at day 14 after fracture where the KEGG pathway “adrenergic signaling” was differentially expressed. The genes α_1D_ and β_2_ were significantly upregulated, giving rise to its potential role in delayed fracture healing, whereas no differences in α_1_ and β_2_ receptor protein expression levels could be detected (Acker, 2025). In addition to our RNA-seq data, other previous studies also showed that adrenergic signaling might play a role in the pathomechanism of osteoporosis (Ma et al., 2013). Osteoporotic patients display a chronic low-grade inflammatory phenotype due to altered cytokine expression and immune-cell profile (Romas and Martin, 1997; Breuil et al., 2010; Fischer et al., 2018). Furthermore, osteoporotic bone is associated with heightened sympathetic tone after fracture, resulting in accelerated bone loss and impaired healing (Huang et al., 2024). Based on these findings, we hypothesized that OVX mice will display increased catecholamine levels. However, this was not verified by our data. Systemic blood catecholamine measurement revealed higher adrenaline levels and significantly increased levels of noradrenaline and dopamine in pre-fracture sham mice compared to OVX mice. Additionally, 3-days post-fracture sham mice also displayed increased adrenaline levels in the blood plasma. Furthermore, phentolamine treatment led to increased noradrenaline levels in sham mice, while adrenalin levels were increased in OVX mice, which might be because of a feedback loop upon alpha receptor blockade. Only adrenaline levels in the bone marrow of the unfractured femur at 21 days after surgery were increased in OVX mice. Unfortunately, based on the current knowledge, there is no explanation for this contradictory finding, warranting further studies.

Clinically, adrenoceptor blockers such as the unspecific β-blocker propranolol are mainly used for the treatment of cardiovascular diseases, but little is known about their impact on fracture healing, especially when administered in the early inflammatory phase of fracture healing. We performed flow cytometry analysis with early fracture hematoma to understand the effect of different adrenoceptor blockers on immune-cell recruitment. Our results indicate that phentolamine (α-receptor blockade) had the most prominent effects on immune-cell recruitment at 1 day after fracture. Especially, B-cell and T-cell recruitment was increased in sham phentolamine-treated mice. However, the most interesting finding is the increase in Ly6G^+^ cells upon propranolol and butoxamine treatment, suggesting an overshooting immune cell activation after the blockade of β-adrenergic signaling. This is in direct contrast to our own previous findings where we observed that propranolol treatment directly before fracture surgery could reduce the numbers of neutrophils in the early fracture hematoma (Haffner-Luntzer et al., 2019). However, this earlier study was conducted in male mice and not in female mice, like in the present study. Therefore, unexpectedly, we observed a sex-dependent response, with neutrophil accumulation not being reduced and, in some conditions, even increasing. This divergence suggests that adrenergic control of myeloid cell trafficking is context-dependent and may be modulated by sex hormones and/or hormone-dependent immune set points. Estrogens and progesterone can shape neutrophil production, mobilization, endothelial adhesion molecule expression, and chemokine gradients (Straub, 2007); thus, adrenergic blockade may interact with hormone-regulated pathways to yield qualitatively different outcomes in females compared with males. We, therefore, interpret the present results as evidence that adrenergic signaling is not uniformly pro-inflammatory across sexes but instead integrates with sex-specific endocrine–immune networks.

In contrast to our expectations, the short-term blockade of adrenergic signaling in the early inflammatory phase of fracture healing also did not improve fracture healing outcomes in both osteoporotic and non-osteoporotic female mice. BV/TV and BV were significantly reduced in OVX mice independent of every type of treatment, and the histological evaluation confirmed these data. This might be because the overshooting recruitment of neutrophils to the fracture hematoma in OVX mice could not be rescued by blocker treatment. Therefore, our hypothesis that short-term blockade of adrenergic signaling in the early inflammatory phase of fracture healing is able to block excessive neutrophil recruitment, therefore enhancing fracture healing in OVX mice, was proven wrong. Other studies on rodents treated with beta blockers for a longer period during fracture healing showed different results. Huang et al. (2024) reported that long-term blockade of β-adrenergic signaling with propranolol and in combination with parathyroid hormone (PTH) led to enhanced bone formation and fracture healing in osteoporotic models, suggesting that excessive adrenergic signaling can be detrimental in this context (Huang et al., 2024). On the other hand, traumatic brain injury (TBI) is associated with a hyperadrenergic state (Hinson and Sheth, 2012; Qian et al., 2022) and increased β_2_-adrenergic signaling, thereby enhancing fracture healing in mice, while TBI in combination with propranolol treatment resulted in impaired fracture healing (Jahn et al., 2024). In addition, Liu and colleagues reported that a chemical sympathectomy blocks TBI-induced accelerated fracture healing by preventing the shift to an anti-inflammatory environment in the bone marrow of mice (Liu et al., 2023). Furthermore, sensory and sympathetic nerve fibers (SNF) are crucial for proper callus formation since the loss of SNF leads to an induction of bone resorption (Niedermair et al., 2020; Niedermair et al., 2014). Taken together, these studies and our data suggest that adrenergic signaling plays a pivotal role in the fracture healing process, but its role appears to be highly dependent on the target cells, comorbidities, and, most likely, sex.

One limitation of our study is that we only used female mice, which can be explained by our research goal of investigating the effects of adrenoceptor blockers on fracture healing in postmenopausal mice. In addition, only one dosage of each blocker with only one subcutaneous injection per day was studied. In addition to that, the half-life of the blockers varies markedly, from 20 min for phentolamine to 3–4 h for propranolol, while no data are available for butoxamine, as it is used exclusively for research purposes. These differences might result in fluctuating levels throughout the day. Nevertheless, the treatment regime was chosen based on the available literature.

In conclusion, we were able to demonstrate that the early blockade of adrenergic signaling did not improve fracture healing in both osteoporotic and non-osteoporotic bone. In contrast to previous studies with male mice, propranolol and butoxamine treatment increased neutrophil numbers in the early fracture hematoma in female mice, indicating an overshooting immune response and a sex-dependent effect of adrenoceptor blocker treatment. Further research is needed to better understand the sex-specific and cell-specific effects of adrenergic signaling on fracture healing.

## Data Availability

The datasets presented in this study can be found in online repositories. The names of the repository/repositories and accession number(s) can be found below: https://doi.org/10.1002/jbmr.4797.
