# Supplementary material for: The influence of adrenoceptor blocker treatment on fracture healing in osteoporotic and non-osteoporotic bone
Source: Front Physiol. 2026 Feb 16;16:1726583. doi: 10.3389/fphys.2025.1726583 (PMC12950559; doi:10.3389/fphys.2025.1726583)
Supplement: Supplementary file 1 [file DataSheet1.docx]

**Supplemental**

**
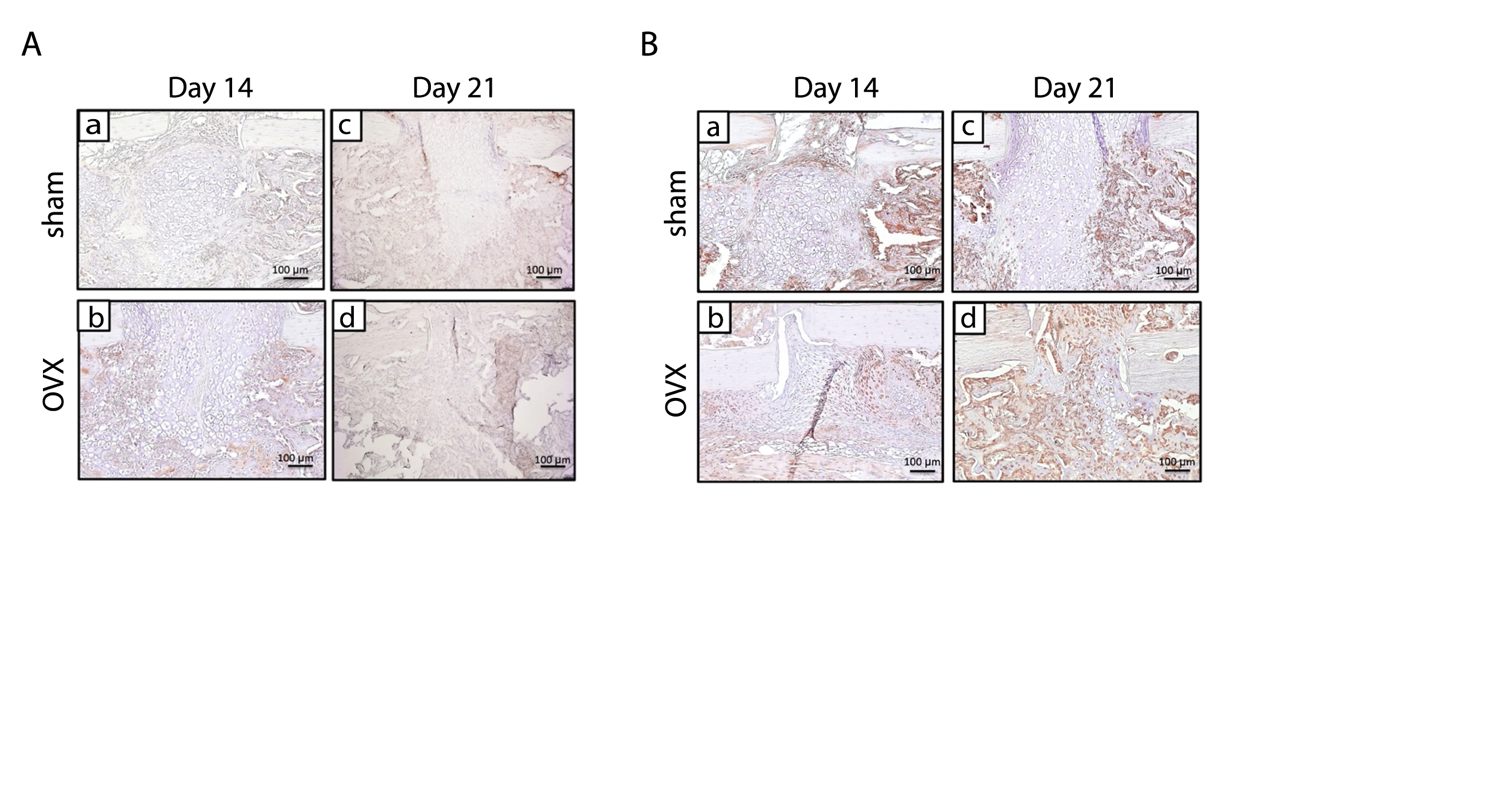
**

**Supplementary Figure 1: A:** α_1_ IHC staining in the fracture callus 14- and 21-days post-injury. No significant differences between sham and OVX mice. (Day 14: P=0.55; Day 21: P=0.29) **B:** β_2_ IHC staining in the fracture callus 14- and 21-days post-injury. No significant differences between sham and OVX mice. (Day 14: P=0.16; Day 21: P=0.79) [29] Statistical significance was determined by unpaired t-test (comparison of sham vs. OVX per timepoint). (*N*=4-6)

**
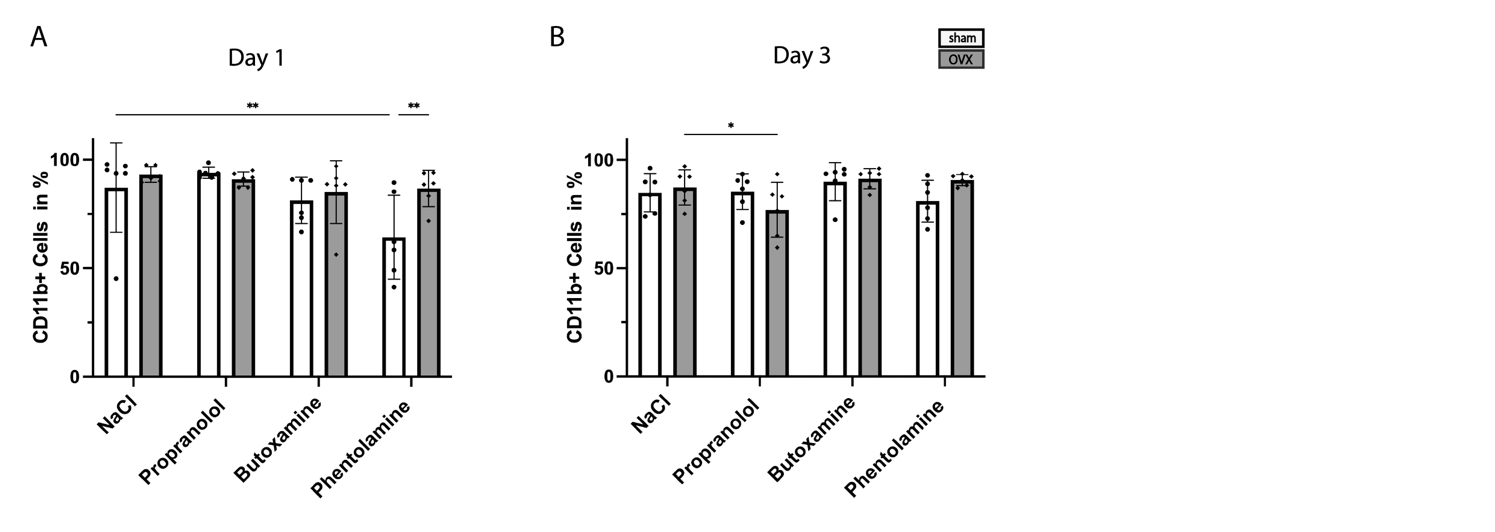
**

**Supplementary Figure 2:** Percentage of CD11b+ cells of alive population; Parent population of Ly6G+ and F4/80+ cells. Statistical significance was determined by Two-way ANOVA with Fisher LSD test. *P < 0.05, **P < 0.01, ***P < 0.001, ****P < 0.0001. (*N*=5-8)

**
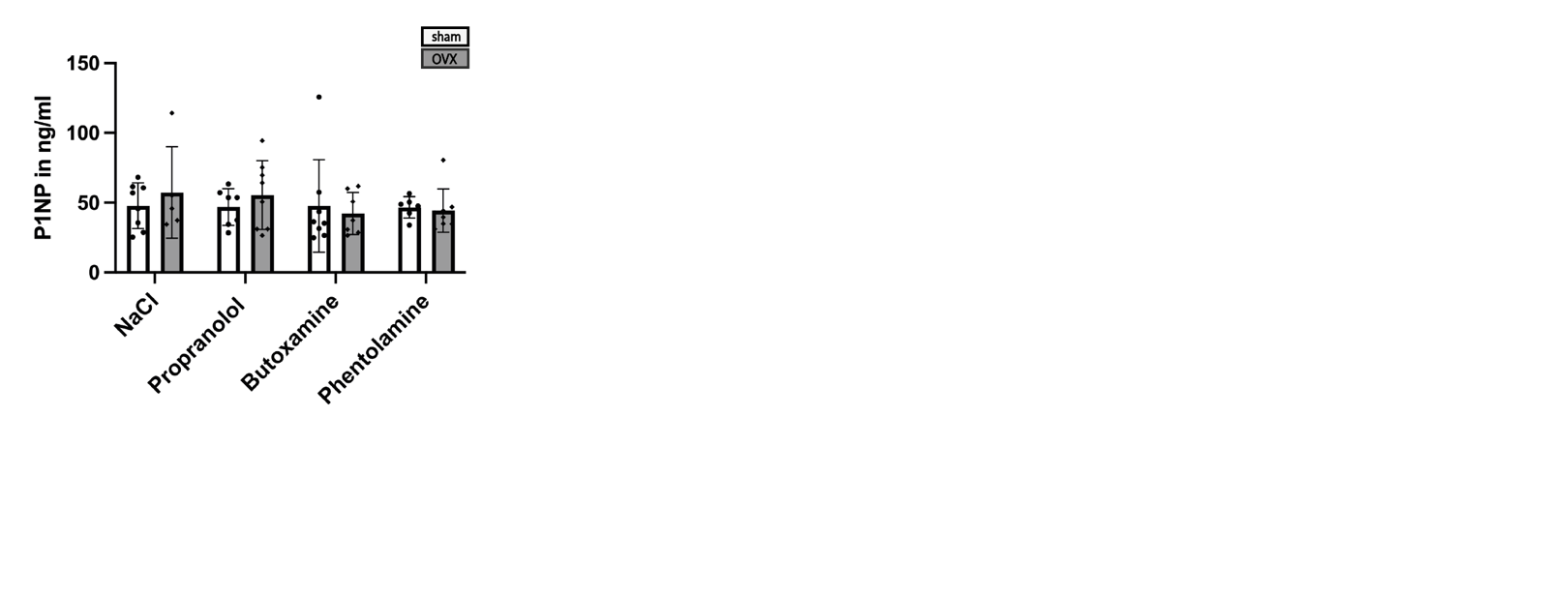
**

**Supplementary Figure 3:** P1NP ELISA 21 days post-fracture. No significant differences between sham and OVX as well as treatments. Statistical significance was determined by Two-way ANOVA with Fisher LSD test. *P < 0.05, **P < 0.01, ***P < 0.001, ****P < 0.0001. (*N*=5-8)
